# First report of *Mesocriconema sphaerocephalum* (Taylor, 1936) Loof, 1989 associated with carrot (*Daucus carota* subsp. Stativus) in Vietnam

**DOI:** 10.21307/jofnem-2019-048

**Published:** 2019-07-23

**Authors:** Thi Duyen Nguyen, Huu Tien Nguyen, Thi Mai Linh Le, Thi Tuyet Thu Tran, Neriza Nobleza, Quang Phap Trinh

**Affiliations:** 1 Institute of Ecology and Biological Resources, Vietnam Academy of Sciences and Technology, 18 Hoang Quoc Viet, Cau Giay, 100000 Hanoi, Vietnam.; 2 Graduate University of Science and Technology, Vietnam Academy of Sciences and Technology, 18 Hoang Quoc Viet, Cau Giay, 100000 Hanoi, Vietnam; 3 Faculty of Environmental Sciences, VNU University of Science, 334 Nguyen Trai, Hanoi, Vietnam; 4 College of Agriculture, Mindanao State University, Main Campus, Marawi City, 9700, Lanao del Sur, The Philippines

**Keywords:** Carrot, Criconematidae, *Daucus carota* subsp, Stativus, *Mesocriconema sphaerocephalum*, Ring nematode, Vietnam

## Abstract

Our study recorded the presence of *Mesocriconema sphaerocephalum* on carrot in Hanoi city and Hai Duong province in Vietnam. This species was identified by morphometric, morphological characterizations, and molecular characterization of D2D3 of 28S rDNA sequence. To our knowledge, this is the first report of *M. sphaerocephalum* on carrot in Vietnam.

*Mesocriconema* spp. belongs to the family Criconematidae Taylor, 1936 (1914) (Thorne, 1949). Species in this group are known as the ring nematodes, and they are one of important ectoparasitic nematodes that can be a potential threat at high soil population density. Some species of the ring nematodes caused yield loss up to 50% such as *Macroposthonia ornatum* (Raski, 1958) Loof and De Grisse, 1989 in pod field (Siddiqi, 2000).

Our study recorded *Mesocriconema sphaerocephalum* (Taylor, 1936) Loof, 1989 on carrot in Hanoi city and Hai Duong province in Vietnam. *Mesocriconema sphaerocephalum* was found on 10% of 130 soil samples. Density of this nematode was up to 45 individuals/250 g soil. To our knowledge, this is the first report of *M. sphaerocephalum* on carrot.

## Materials and methods

Nematodes were extracted from soil samples using modified Baermann tray method (Whitehead and Hemming, 1965). For morphological characterizations, permanent slides of nematodes were observed through the Carl Zeiss Axio Lab.A1 light microscope. Measurements and pictures were taken using a ZEN lite software on ZEISS Axiocam ERc5s digital camera (Nguyen et al., 2017).

For molecular studies, Primers D2A (5′-ACAAGTACCGTGGGGAAA GTTG-3′) and D3B (5′-TCGGAAGGAACCAGCTAC TA-3′) were used to amplify D2D3 of 28S rDNA region (Nguyen et al., 2017). Obtained sequence was used for a Blast search in GenBank (Altschul et al., 1997). The data set was analyzed using maximum likelihood (ML) method in MEGA 6 program with 1,000 bootstrap replications. The best fit model of DNA evolution was obtained using the Model test in MEGA 6 (Nguyen et al., 2017).

## Results and discussion

### Morphological characterization

**Figure 1:**
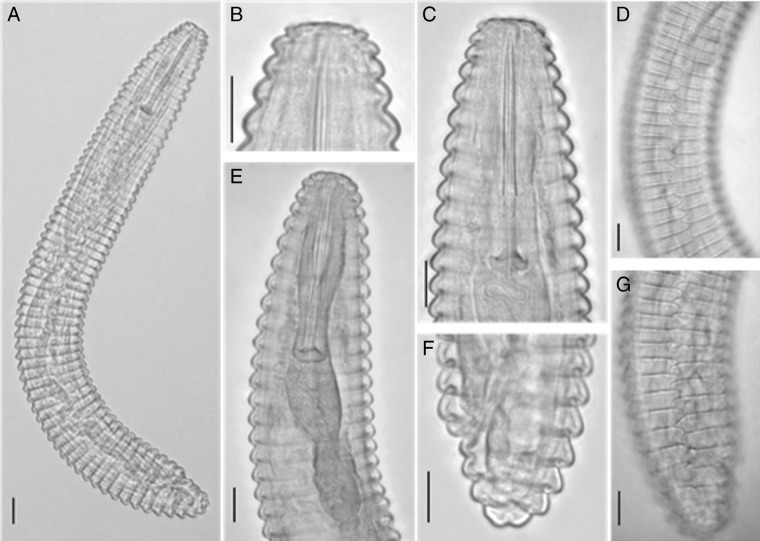
*Mesocriconema sphaerocephalum* on carrot in Vietnam. (A) entire body; (B) head region; (C) stylet; (D) anastomoses at mid-body; (E) pharyngeal region; (F) tail region; (G) anastomoses at tail region (Scale bar: 10 µm).

Measurements of *M. sphaerocephalum* in this study are in agreement with measurement of *M. sphaerocephalum* in Geraert (2010) (Table 1). Females of *M. sphaerocephalum* on carrots are characterized by the following traits: body curved ventrally (Fig. 1A); lip region bearing two annuli with flattened labial disc (Fig. 1B); first body annulus much smaller than second one with smooth edge, sloping posteriorly (Fig. 1B); cuticle annuli at mid-body 4 to 5 µm wide; anastomoses numerous at lateral field, forming zigzag lines (Fig. 1D,G); stylet robust, knobs 9 to 10 µm wide (Fig. 1C); vulva located near posterior end; tail rounded (Fig. 1F).

**Table 1.** Measurements of females of *M. sphaerocephalum* on carrot in Vietnam. All measurements are in µm and in form: mean ± SD (range), except for ratio.

	*M. sphaerocephalum*	
Measurement	Population in Hanoi city	Population in Hai Duong province
n	10	10
L	298 ± 13.8 (274–316)	330.7 ± 14.4 (309.4–360)
a	8.6 ± 0.5 (7.5–9.2)	9.7 ± 0.6 (8.8–10.6)
b	2.8 ± 0.1 (2.5–3)	3.2 ± 0.2 (3-3.5)
c	44 ± 9.8 (31–65)	52 ± 9.2 (39–69)
c′	0.4 ± 0.1 (0.3–0.5)	0.4 ± 0.1 (0.3–0.5)
VL/VB	0.8 ± 0.1 (0.7–1)	0.7 ± 0.1 (0.6–1)
V	94 ± 1 (93–96)	95 ± 0.4 (94–95)
Stylet length	55 ± 1.6 (53–58)	51 ± 1.5 (48-54)
Anterior end to nerve ring	82 ± 2.4 (78–85)	76 ± 2.6 (72–82)
Anterior end to end of pharynx	107 ± 2.7 (104–110)	103.3 ± 3 (98–109)
Anterior end to secretory-excretory pore	110 ± 4 (105–117)	108 ± 2.3 (104-113)
Max. body diam.	34.8 ± 1 (33–37)	34 ± 1.4 (31–37)
Body diam. at vulva	23.2 ± 1.4 (21–25)	24.3 ± 1.7 (22.2–28)
Body diam. at anus	17.7 ± 1.8 (14.3–19.4)	18 ± 2 (14.2–22)
Tail length	7 ± 1.3 (4.4–8.8)	6.6 ± 1.2 (4.8–9)
Rst	12.4 ± 0.5 (12–13)	12 ± 0.3 (12–13)
Roes	20.7 ± 0.8 (20–22)	20.4 ± 0.6 (20–22)
Rex	21.5 ± 0.7 (20–22)	21.6 ± 0.5 (21–22)
Rv	4.8 ± 0.4 (4–5)	4.3 ± 0.4 (4–5)
Ran	2.2 ± 0.4 (2–3)	2 ± 0.3 (2–3)
Rvan	3 ± 0.3 (2–3)	2.2 ± 0.4 (2–3)
R	65 ± 1.6 (63–68)	64 ± 1.4 (62–66)

### Molecular characterization

**Figure 2:**
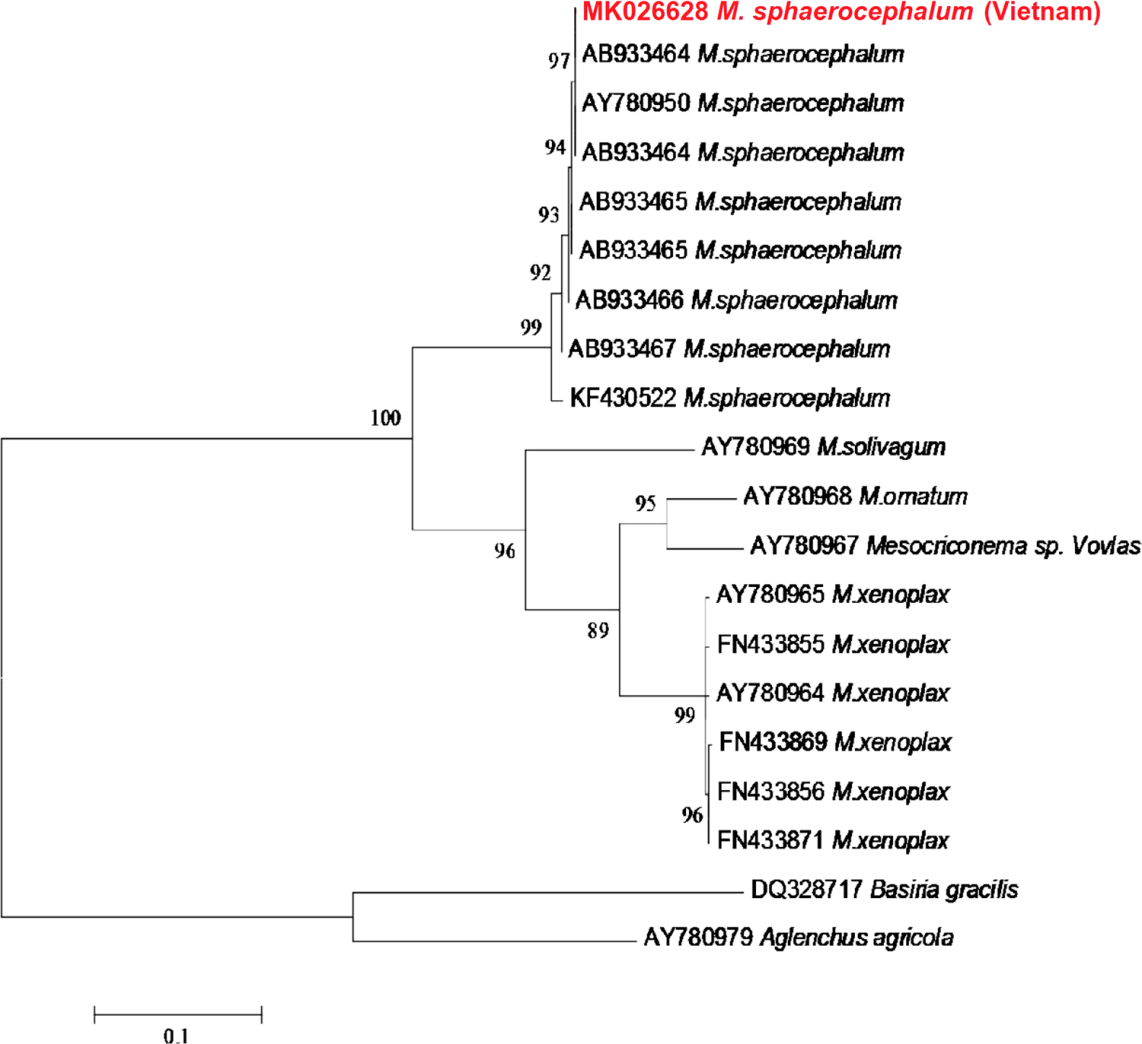
Phylogenetic tree generated from D2D3 of 28S rDNA sequences based on ML method (TN93 + G model) with 1,000 replications. Sequence of *Mesocriconema sphaerocephalum* on carrot in Vietnam is in red.

D2D3 of 28S rDNA sequence of *M. sphaerocephalum* in this study was 730 bp, submitted to GenBank under accession number: MK026628. It is 99% similar to *M. sphaerocephalum* (AB933464) in GenBank. This sequence varied 0 to 2% compared to other sequences of *M. sphaerocephalum* in GenBank. The maximum likelihood phylogenetic tree placed sequence of *M. sphaerocephalum* on carrots together with other sequences of *M. sphaerocephalum* from GenBank (Fig. 2).
